# Gaming Disorder Seen Through the Prism of Dual Diagnosis: Prevalence and Associated Factors

**DOI:** 10.3389/fpsyt.2022.821432

**Published:** 2022-07-08

**Authors:** Malcolm Barrangou-Poueys-Darlas, Clémence Cabelguen, Vincent Garrouste, Juliette Leboucher, Bruno Rocher, Gaëlle Challet-Bouju, Marie Grall-Bronnec

**Affiliations:** ^1^Nantes Université, CHU Nantes, UIC Psychiatrie et Santé Mentale, Nantes, France; ^2^CHU Tours, Emergency Department, Tours, France; ^3^Nantes Université, University of Tours, CHU Nantes, CHU Tours, INSERM, Methods in Patients Centered Outcomes and Health Research, SPHERE, Nantes, France

**Keywords:** dual diagnosis, gaming disorder, addiction, associated factors, risk of suicide

## Abstract

**Introduction:**

Dual diagnosis (DD) is defined as the co-occurrence of at least a psychiatric disorder and at least an addictive disorder. Most studies about DD considered substance use disorders. In 2018, gaming disorder (GD) was recognized as a formal disorder and integrated into the category of addictive disorders in the 11th version of the International Classification of Diseases. Our objectives were to measure DD prevalence among GD patients and to assess factors associated with the presence of DD.

**Methods:**

As part of the EVALuation of behavioral ADDictions (EVALADD) cohort, 92 patients with GD were included in the present study. Psychiatric disorders, including anxiety, mood, and psychotic disorders, were explored with the Mini International Neuropsychiatric Interview (MINI 5.0.0). Probable adult attention-deficit/hyperactivity disorder (ADHD) was screened with the Wender Utah Rating Scale (WURS) in childhood and with the ADHD Self-Report Scale-V1.1 (ASRS) in adulthood. Finally, personality was assessed using the 125-item version of the Temperament and Character Inventory (TCI-125), motives for gaming with the Videogame Motives Questionnaire (VMQ) and attachment styles with the Relationship Scales Questionnaire (RSQ). To measure the prevalence of DD among GD patients, we considered the occurrence of current GD with current anxiety, mood, or psychotic disorders, or with probable current ADHD. We also performed a multivariate analysis to identify independent factors associated with DD.

**Results:**

More than half (55.4%) of GD patients suffered from DD. We found a high prevalence of probable ADHD (38%) and anxiety disorders (29% suffering from generalized anxiety disorder, social, agoraphobia or panic disorder). Four variables were significantly associated with DD: suicidal thoughts [odds ratio (OR) = 6.83, 95% confidence interval (95%CI) (1.66–28.09)], VMQ “coping” scores [OR = 1.18, 95%CI (1.01–1.38)], TCI-125 “harm avoidance” scores [OR = 1.04, 95%CI (1.01–1.07)] and “novelty seeking” scores [OR = 1.03, 95%CI (1.00–1.06)].

**Discussion:**

The prevalence of certain psychiatric disorders among GD patients far exceeded that observed in the general population. Both ADHD and suicidal ideations should particularly be screened among GD patients. Specific interventions targeting personality dimensions associated with DD but also on the management of negative affect should represent new treatment opportunities.

## Introduction

Since 1983, some authors have noticed “obsessive” behaviors among video game players (1). Thirty years later, “Internet Gaming Disorder” first appeared in section III of the Diagnostic and Statistical Manual fifth edition (DSM-5) (2). Finally, in May 2018, after many debates, the World Health Organization (WHO) recognized gaming disorder (GD) as a formal addictive disorder in the 11th International Classification of Diseases (ICD-11), given the common characteristics with the other addictive disorders already included (3). According to the ICD-11, the three core symptoms considered for a diagnosis of GD are impaired control over gaming, increasing priority to gaming over other activities and continuation of gaming despite negative consequences. These symptoms must occur for at least 12 months and result in a significant impairment in important areas of functioning.

Multiple mechanisms are involved in the initiation and persistence of GD, such as motivations to play and escapism (4–6), attachment style (7, 8) and certain psychopathological traits, such as impulsivity and poor emotion regulation (9). Numerous studies have reported links between GD and comorbid psychiatric disorders including anxiety disorders, depressive disorders, attention-deficit/hyperactivity disorder (ADHD), conduct disorder, substance use disorders (SUDs) and pathological personality traits (10–14). Associated disorders can be a cause or consequence of GD, but the association can also form a complex clinical entity.

The term “dual diagnosis” (DD) describes the co-occurrence of a SUD and a psychiatric disorder (15) while the term “dual disorder” illustrates a new disorder including an addictive disorder and another psychiatric disorder. This combination creates a new pathology that is more complex than the simple summation of the two disorders. Studying the links between addictive and other psychiatric disorders is of growing interest due to the prevalence and gravity of these situations. DD patients have less favorable prognoses with more severe symptoms for each of the disorders and greater chronicity (15–17). DDs are mostly studied *via* specific associations such as cannabis consumption and psychosis or alcohol consumption and mood disorders, but the mechanisms shared by all types of DD remain incompletely understood. Previous studies found that patients with DD were more likely to be men, be young, and have a history of aggression (18). A recent comprehensive review and meta-analysis comparing personality traits between patients suffering from psychotic disorders with and without comorbid SUDs found impulsive and externalizing trait personality domains unique to the DD group (19).

Although DD has mostly been studied through its association with a SUD, studying the applicability of DD to all addictive disorders, including behavioral addictions, would allow a better understanding of behavioral addictions. The existence of many different combinations of addictive and psychiatric disorders support this hypothesis (20). In particular, due to the recent inclusion of GD in the framework of disorders due to substance use or addictive behaviors, it seems relevant to study whether specific psychopathological or clinical features, known to be associated with GD, might also be differentially associated with DDs involving GD.

We made the assumption that patients suffering from GD have frequent co-occurrent psychiatric disorders, and that they do not constitute a homogeneous clinical group. It would be important to differentiate the management of those suffering from an isolated GD from those suffering from a DD. Thus, our main objective was to determine the prevalence of DD among GD patients at the beginning of treatment. We also aimed to identify characteristics associated with DD among GD patients.

## Materials and Methods

### Procedure

Data for this study were extracted from the EVALuation of behavioral ADDictions (EVALADD) cohort (NCT01248767). The EVALADD cohort involves a prospective follow-up of outpatients over 15.25 years old (the threshold that separates pediatric and adult care in our hospital) from the initiation of specific care for a behavioral addiction at Nantes University Hospital, France. Only patients who provided their written informed consent (including consent from parents or guardians for participants under age 18) were included in the EVALADD cohort, and patients with cognitive impairment or difficulties reading or writing French were not included. The EVALADD procedure includes several repeated assessments conducted at the initiation of addiction treatment, after 6 months, after 1 year and then after each subsequent year as long as the patient agrees to complete the follow-up. The assessments are based on a multiaxial psychological assessment performed through a face-to-face structured interview and self-administered questionnaires. The structured interviews were conducted by trained and qualified research staff with experience with behavioral addictions.

### Participants

**Figure 1:**
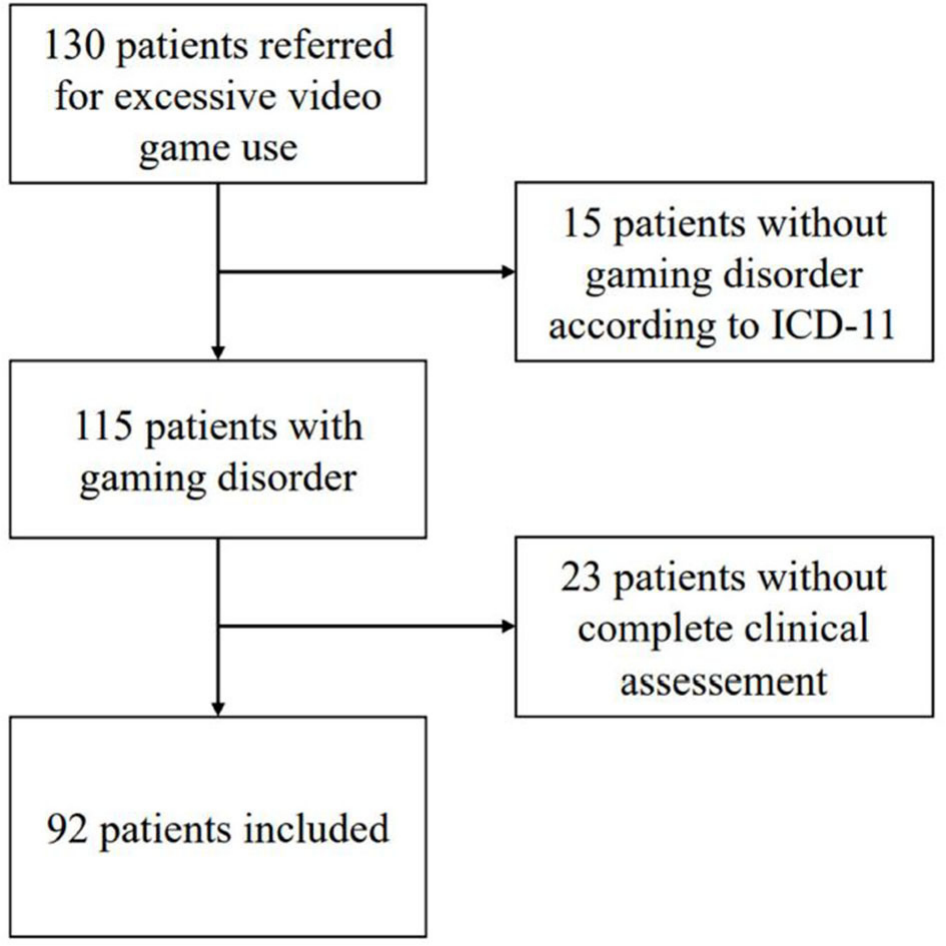
Flow chart of patients participating in the study from August 2012 to August 2020.

For the present study, we selected only 130 outpatients (the sample is composed of an adolescent and adult population) who were referred for excessive video game use (according to the patient himself or herself or according to his or her relatives) from August 2012 to August 2020. After exclusion of those who did not match the GD ICD-11 criteria or those for whom data were incomplete, 92 patients were included in the analysis (Figure 1). We used only data collected at the initiation of addiction treatment.

### Measures

#### Gaming

As the ICD-11 criteria had not yet been published at the time of the study, a thorough investigation of the medical records of the 130 identified patients allowed us to search retrospectively for the presence or absence of the three ICD-11 criteria during the 12 months preceding inclusion, and for evidence of functional impairment. Inclusion in the study required satisfying the 3 criteria and having a functional impairment that led to confirmation of the diagnosis of GD. All patients' medical records were analyzed by a researcher and the referred psychiatrist in a double-blind manner.

We used the Videogame Motives Questionnaire (VMQ), derived from the Gambling Motives Questionnaire (GMQ) (21), to evaluate patients' motives for gaming. The only adaptation was to switch “gambling” to “gaming”. This questionnaire assesses three dimensions: coping, enhancement, and social motivation. Higher scores for a particular type of motivation corresponds to higher levels of motivation in that dimension. It should be noted that there are no cut-off scores and that this questionnaire results in a profile of the motivations for playing.

#### Other Psychiatric Disorders

DD status in our study was defined as the association of GD and at least one current psychiatric disorder at inclusion.

We used the Mini International Neuropsychiatric Interview version 5.0.0 (MINI) (22) to diagnose the following current psychiatric disorders: depressive episodes, dysthymia, manic or hypomanic episodes, panic disorder, agoraphobia, social phobia, obsessive-compulsive disorder, post-traumatic stress disorder, psychotic disorders and generalized anxiety. Furthermore, current SUDs were assessed, even if they were not considered for the definition of DD.

The EVALADD cohort did not include a formal ADHD diagnosis but probable ADHD was explored through two screening questionnaires. Probable ADHD symptoms in childhood were explored by the Wender Utah Rating Scale-Child (WURS-C) (23), and symptoms in adulthood were explored by the Adult Attention Deficit Hyperactivity Disorder Self-Report Scale Screener v1.1 (ASRS-1.1) (24, 25). Based on the results of these questionnaires, it was possible to screen for the presence of current probable ADHD among minor patients (WURS-C score ≥ 46/100) and among adult patients (WURS-C score ≥ 46/100 and a positive screening with the ASRS Screener v1.1).

Furthermore, suicidal ideation was assessed by a specific section of the MINI.

#### Psychopathology

The Relationship Scales Questionnaire (RSQ) (26) is one of the most commonly used questionnaires for assessing different attachment styles. We calculated the weighted average of the scores for each attachment dimension and thus determined the patient's predominant attachment style.

The Temperament and Character Inventory−125 items (TCI-125) (27, 28) is a self-administered questionnaire providing a personality profile. It measures seven dimensions including four temperaments (“novelty seeking,” “harm avoidance,” “reward dependence,” and “persistence”) and three character traits (“self-directedness,” “self-cooperation,” and “self-transcendence”). All items are coded as true or false, with the attribution of 0 or 1 point based on the item. For each dimension, the score is calculated by the following formula to obtain a standardized mean: sum of the score of the items^*^100/number of items of the dimension. Scores for each dimension could range from 0 to 100.

### Statistical Analysis

Statistical analyses were performed using STATA 11^®^ v 11.2 (Statistical Data Analysis/TX/USA) software.

First, a descriptive analysis of the characteristics of our population was carried out, and DD prevalence among the GD patients was calculated. These data are presented as means for continuous variables and percentages for categorical variables. We tested the equality of variances and the normality of the distributions in the comparative analyses.

Then, univariate analyses were conducted comparing results between the patients suffering from isolated GD and those suffering from DD. For categorical variables, a Chi^2^-test was used when possible or Fisher's exact-test if not. For continuous variables, we used Welch's parametric *t*-test (if variance equality was rejected) or the non-parametric Mann-Whitney test, with a *p*-value ≤ 0.05 defining a significant difference between the two groups.

Thereafter, a multivariate logistic regression analysis was performed using an iterative selection procedure to select the variables that were significantly associated with “DD” status, as assessed by likelihood ratio tests. Variable candidates for the model were those associated with “DD” in the univariate analyses with a *p*-value < 0.20 (29). Then, backward selection was applied using the *p*-value < 0.05 criterion. The corresponding odds ratio (OR) and associated 95% confidence interval (95% CI) were estimated. The ability of the final model to discriminate between the presence or absence of a DD was assessed using the area under the receiver operating characteristic (ROC) curve, and the goodness-of-fit of the model was assessed using the Hosmer-Lemeshow test.

### Ethics

The EVALADD cohort study was conducted in accordance with the Good Clinical Practice Guidelines and the Declaration of Helsinki, with approval from the local ethics committee (Groupe Nantais d'Ethique dans le Domaine de la Santé, GNEDS, Nantes) on September 6, 2012 and amended by the Research Ethics Committee (CPP Ile de France VI) on August 3, 2018.

## Results

### Description of the Sample

The results are shown in Table 1. A large majority of our patients were young men, single, unemployed, with a family history of addictive disorders. Ages ranged from 15 to 72 years, with a median age of 22. Suicidal ideation was reported by 28% of the sample.

**Table 1:** Sample description and univariate analyses by the presence or absence of dual diagnosis among gaming disorder patients (*N* = 92).

	**Variable [mean (SD) or *n* (%)]**	**Total (*n* = 92)**	**Isolated GD (*n* = 41)**	**DD (*n* = 51)**	***p-*value**
Sample characteristics	Age	24.6 (±9.67)	24.7 (±11.0)	24.5 (±8.59)	0.93
	Male	84 (91%)	38 (93%)	46 (90%)	0.73
	Single	78 (85%)	34 (83%)	44 (86%)	0.66
	Professional activity^*^	16 (17%)	12 (29%)	4 (7.8%)	0.01
	Family history of addictive disorder^*^	56 (61%)	19 (46%)	37 (73%)	0.01
	Recent suicidal ideation^*^	25 (27%)	4 (9.8%)	21 (41%)	0.001
VMQ	Coping^*^	15.3 (±3.54)	13.8 (±3.64)	16.5 (±3.01)	0.01
	Enhancement^*^	15.0 (±3.11)	14.2 (±3.05)	15.6 (±3.06)	0.07
	Social^*^	8.87 (±2.94)	8.10 (±2.29)	9.49 (±3.27)	0.01
RSQ	Predominantly insecure attachment style^*^	72 (78%)	28 (68%)	44 (86%)	0.04
TCI-125	Temperament harm avoidance^*^	57.3 (±24.6)	47.1 (±22.9)	65.6 (±22.8)	0.01
	Temperament novelty seeking^*^	54.2 (±21.6)	50.9 (±19.4)	57.0 (±23.1)	0.17
	Temperament persistence	33.0 (±28.8)	33.7 (±28.1)	32.5 (±29.7)	0.85
	Temperament reward dependence	51.2 (±18.2)	50.7 (±19.1)	51.6 (±17.6)	0.70
	Character self-cooperation^*^	68.9 (±19.0)	71.8 (±18.7)	66.5 (±19.1)	0.15
	Character self-directedness^*^	45.4 (±18.4)	52.6 (±17.2)	39.6 (±17.4)	0.01
	Character self-transcendence^*^	30.4 (±22.7)	26.5 (±19.8)	33.6 (±24.4)	0.13

*DD, dual diagnosis; GD, gaming disorder; RSQ, Relationship Scales Questionnaire; TCI-125, Temperament and Character Inventory−125; SD, standard deviation; VMQ, Videogame Motives Questionnaire (from Gambling Motives Questionnaire).*

* *Variables included in the initial multivariate model (p-value < 0.20)*.

Moreover, current SUDs were diagnosed, with 30 patients (32.6%) for tobacco, 10 patients (10.9%) for alcohol, 9 patients (9.8%) for cannabinoids, and 0 patient for other illicit substances. Finally, four patients (4.3%) also suffered from current gambling disorder.

### Prevalence of Dual Diagnosis

Out of 92 patients, 51 (55%) suffered from DD: 22 DD were characterized by multiple current psychiatric disorders and 29 by a single psychiatric disorder. Of the 29 patients with a single current comorbid psychiatric disorder, 18 suffered from probable ADHD, 9 from an anxiety disorder (generalized anxiety disorder, social phobia, agoraphobia, or panic disorder) and 2 from a mood disorder (major depressive episode, manic episode, or dysthymia). The description of associated disorders is available in Table 2.

**Table 2:** Prevalence of associated disorders (*N* = 92).

	***n* (%)**
Dual diagnosis	51 (55%)
Probable ADHD	35 (38%)
Generalized anxiety disorder	14 (15%)
Social phobia	12 (13%)
Major depressive episode	12 (13%)
Agoraphobia	10 (11%)
Post-traumatic stress disorder	3 (3%)
Obsessive compulsive disorder	2 (2%)
Manic episode	2 (2%)
Panic disorder	2 (2%)
Psychotic disorders	1 (1%)
Dysthymia	0 (0%)

*ADHD, attention-deficit/hyperactivity disorder*.

### Factors Associated With Dual Diagnosis

**Figure 2:**
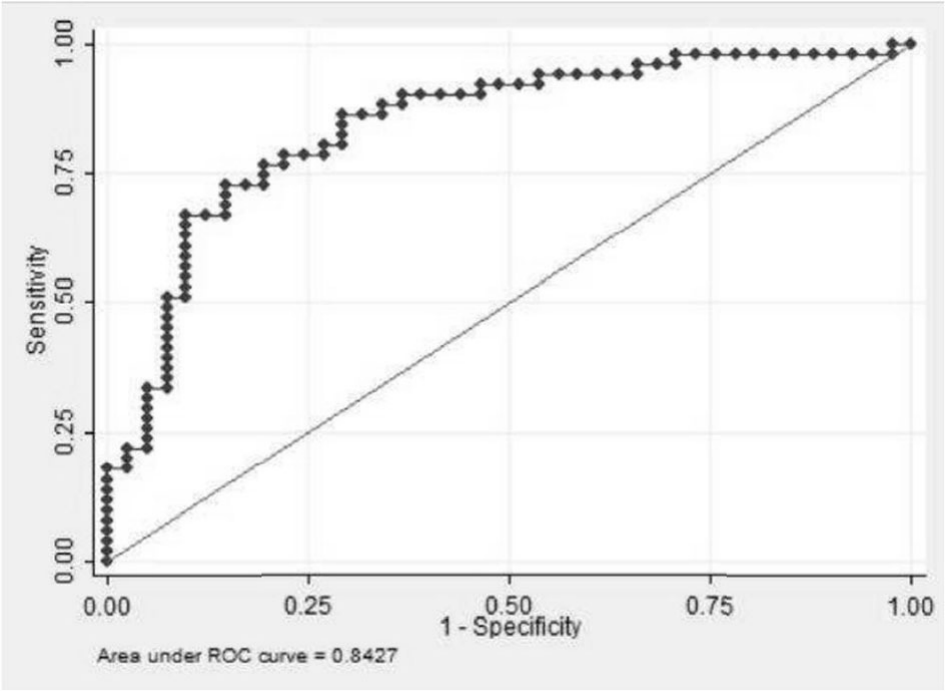
Receiver operating characteristic curve of the final model.

The results of the comparison between “Isolated GD” and “DD” are provided in Table 1. Among the 17 variables of interest, 12 were associated with DD at a 0.20 level of significance: the presence of professional activity, presence of family history of addictive disorder, recent suicidal thoughts, the three VMQ dimensions (Coping, Enhancement and Social), RSQ predominant insecure attachment style, and five TCI-125 dimensions (harm avoidance, novelty seeking, self-cooperation, self-directedness, and self-transcendence). These variables were then entered as candidates in the multivariate regression, from which four variables were found to be independently associated with DD in the final model (Table 3). Recent suicidal thoughts [OR = 6. 83, 95% CI (1.66–28.09), *p* = 0.008], TCI-125 “novelty seeking” scores [OR = 1.03, 95% CI (1.00–1.06), *p* = 0.029], TCI-125 “harm avoidance” scores [OR = 1.04, 95% CI (1.01–1.07), *p* = 0.002] and VMQ “coping” scores [OR = 1.18, 95% CI (1.01–1.38), *p* = 0.042] were factors associated with the presence of a DD. The Hosmer-Lemeshow goodness-of-fit test showed that the final model was well calibrated, with *p* = 0.87 (*p*-value > 0.05 indicates good model fit), and the area under the ROC curve was 0.84 (0.76–0.92) (Figure 2), showing that the model discriminated well between patients with “Isolated GD” and patients with “DD”.

**Table 3:** Multivariate analysis of factors associated with dual diagnosis among gaming disorder patients (*N* = 92).

**Variable**	**Odds ratio**	**95% Confidence interval**	***P-*value**
TCI-125 novelty seeking	1.03	(1.00–1.06)	0.029
TCI-125 harm avoidance	1.04	(1.01–1.07)	0.002
VMQ coping	1.18	(1.01–1.38)	0.042
Recent suicidal ideation	6.83	(1.66–28.09)	0.008

*TCI-125, Temperament and Character Inventory−125; VMQ, Videogame Motives Questionnaire.*

*Global fitness was assessed using the Hosmer-Lemeshow goodness-of-fit test, with a p-value > 0.05 (0.87), indicating that the model was well calibrated*.

## Discussion

### Main Results

Our observational study of GD patients seeking treatment aimed to determine DD prevalence and to identify characteristics associated with DD. More than half of the sample had GD associated with at least another current psychiatric disorder. Compared to those found in studies on other behavioral addictions, especially gambling disorder, this rate might seem low. For example, in a previous publication, we reported that 75% of our sample of pathological gamblers seeking treatment suffered from at least one psychiatric comorbidity (30). However, we must keep in mind that we only considered current psychiatric disorders in the present study, rather than lifetime disorders, as is the case in many studies. In addition, patients with GD are relatively young, as confirmed in our study, which may limit the possibility to observe the occurrence of a psychiatric disorder. We showed that GD patients were more frequently affected by psychiatric disorders than the general population (31) and that the proportion of DD was quite similar when considering GD (55% in our study) or SUDs, with almost 1 out of 2 patients (32). The main comorbid disorders were essentially anxiety disorders and probable ADHD, with a prevalence of ADHD symptoms almost 10 times higher than that observed in the general population (33). This result is consistent with the literature (11, 14, 34). The high proportion of probable ADHD found in our population may also be explained by certain common characteristics found with patients suffering from gaming disorder such as high impulsivity. Patients with ADHD may find a form of self-medication in playing certain types of video games as a coping mechanism (35). This hypothesis could open up new therapeutic options. ADHD is already known to be highly comorbid with SUDs, and additional comorbid disorders are more frequent among SUDs patients with ADHD than those without ADHD (36). Validating the close links between probable current ADHD and addictive disorders, in this case GD, reinforces the hypothesis of the applicability of the DD concept to addictions in general and to GD more specifically. Regarding comorbid anxiety disorders, several studies have described not only associations between anxiety disorders and GD but also predictive relationships between gaming and depression, anxiety and social phobia with increased levels of these disorders among GD patients and lower levels among patients who ceased gaming (9, 37). Studies have suggested that depressive and anxiety symptoms before the COVID-19 pandemic positively predicted videogame use and problematic gaming during the COVID-19 pandemic (38). On the one hand, anxiety symptoms may facilitate the emergence of GD, and on the other hand, gaming can be used to cope with stressful events and negative emotions. This close and dynamic interrelationship can result in the modification of the features of both GD and associated anxiety disorders, potentially resulting in a DD.

We highlighted that some psychopathological and clinical characteristics of GD patients were associated with DD. First, 41% of patients in the DD group reported suicidal ideation in the month preceding the evaluation and/or a history of suicide attempts. In contrast, a French epidemiological study found that 4.7% of 18–75-year-olds reported having thought about suicide in the previous 12 months and that 7.2% had attempted suicide in their life (39). Suicidal risk should be assessed systematically in patients with DD, particularly in the context of GD. Independent of GD, an association between DD and suicidal ideation has been previously reported in the literature (40). DD patients with one disorder attempting to regulate another disorder may more frequently feel desperate as coping mechanisms fail, which may lead to accumulating negative consequences or the two disorders potentiating each other (e.g., withdrawal in depression and GD); these situations could favor the emergence of suicidal ideation. Finally, since there are multiple pathways to care, promoting liaison care between health services is essential, as suicidal ideation may be a trigger for initiating treatment for GD.

Second, “novelty seeking” and “harm avoidance” dimensions were associated with DD. A recent publication described different personality profiles among GD patients based on the number of comorbid disorders (41). As developed by Cloninger, “novelty seeking” corresponds to behavioral activation to rewarding stimuli and signals, whereas “harm avoidance” reflects a behavioral inhibition to signals recognized as punitive or frustrating (27). One might think that such opposite temperaments linked to the same “DD” entity could reflect diversity among this entity. GD patients with a high “novelty seeking” temperament could develop a different type of DD than “harm avoidance” GD patients given the core difference defining these gamers personalities. Thus, different gamer profiles with different personality traits could favor the emergence of particular comorbid psychiatric disorders and thus favor very different DDs. Recently, a study about ADHD symptoms and video game addiction stated that impulsivity appears to be the ADHD symptom most strongly correlated with video game addiction (42). Therefore, both psychiatric disorders and GD reinforce each other depending on the specific patient temperament. A similar idea of subgroups based on different patient characteristics had previously been identified for gambling disorder. According to Blaszczynski and Nower, three main profiles could be distinguished: behaviorally conditioned problem gamblers, emotionally vulnerable problem gamblers associated with premorbid anxiety and depressive disorders, and antisocial impulsive problem gamblers (43). A recent neuroimaging study attempted to distinguish distinct pathways to explain the development of internet gaming disorder (IGD), according to history of childhood ADHD. IGD participants without childhood ADHD exhibited abnormal hyperconnectivity within the default mode network compared with controls, while IGD participants with childhood ADHD showed expanded functional connectivity between the posterior cingulate cortex and cerebellum, a region involved in executive control, suggesting that altered neural system for executive control in ADHD may predispose to the development of IGD (44).

However, further studies are necessary to consolidate an etiological model of GD based on neurobiology and neuroscience.

Third, we highlighted that gaming to reduce or avoid negative emotions, as measured by the VMQ “coping” dimension, was associated with DD. This specific motivation supports the main concept underlying DD, i.e., gaming is used to alter symptoms of an underlying mental disorder that generates negative emotions. Confronted with mental disorders and negative emotions, one could easily engage in specific activities to regulate these emotions. Escapism through video games as a coping mechanism is a known predictive factor of GD (4, 5); however, in contrast to passive escapism where individuals are merely observers, “active escapism” (45) provides the additional opportunity to interact with the environment, which can facilitate affirmation and empowerment. Using video games as a tool to facilitate the regulation of negative emotions can open new therapeutic perspectives with virtual environments. Thus, gaming motives should be considered not only as a vulnerability factor for the development of DD under certain circumstances but also as a way to discover new treatments for such DD.

### Strengths and Limitations

Our study included patient data only at the initiation of addiction treatment for GD in a cross-sectional manner, without taking into consideration the temporal changes in psychopathology. Longitudinal studies could help understand the onset sequence of disorders and could explore causal links in the emergence of DD. In addition, we used screening and not diagnostic tools in the assessment of ADHD. However, we have sought to limit the risk of over-screening by combining two screening tools. Finally, due to the differences in assessment tools (in particular, no use of semi-structured clinical interviews in other studies) and in the evaluation period (in particular, we focused on current disorders in our study), we were unable to compare our results with previous literature regarding clinical and non-clinical populations.

Nevertheless, we were able to collect a large sample of patients with a homogeneous distribution between the “Isolated GD” and “DD” groups. Our clinical sample is typical of patients suffering from GD, namely, outpatients that include a majority of single young men (46). The exhaustive assessment of a large spectrum of psychiatric disorders, the use of recognized and standardized questionnaires and diagnosis interviews, and the use of the new ICD-11 GD criteria are strengths of our study.

### Orientation and Management

Screening patients for the presence of suicidal ideations is a key point, as they represent one aspect of the urgency and gravity of DD and are highly prevalent among GD patients. Probable ADHD, being the most prevalent comorbid disorder identified in our study, must also be systematically screened for and treated if confirmed.

Assessing patients' personality traits, among other personal characteristics, could help manage GD, as these characteristics could be central within a DD, and certain traits might even lead to specific form of a DD. Integrating therapeutic education sessions about the recognition and prevention of psychiatric disorders into the treatment of those with GD would make it possible to prevent the occurrence of these disorders among the most at-risk patients. Implementing prevention efforts and the early recognition of the function of video games as a potentially harmful coping mechanism would be beneficial among patients suffering from mental disorders. Considering GD through the prism of DD helps us understand the mechanisms involved not only in the emergence of GD but also in the development of DD in general. Future prospective studies focused more on the sequence of onset and development of these disorders would be of great help in understanding the more common forms of DD involving GD.

Integrated health care is now recognized by the Substance Abuse and Mental Health Services Administration (SAMHSA) as the reference for treating DD (47) and we should think about implementing these principles in the treatment of GD.

## Data Availability Statement

The datasets presented in this article are not readily available because the raw anonymized data will be made available only if the purpose is consistent with the consent given by participants and in accordance with the legislation in force in France. Requests to access the datasets should be directed to MG-B, marie.bronnec@chu-nantes.fr.

## Ethics Statement

The studies involving human participants were reviewed and approved by Groupe Nantais d'Ethique dans le Domaine de la Santé, GNEDS, Nantes Research Ethics Committee, Comités de protection des personnes (CPP) Ile de France VI. Written informed consent to participate in this study was provided by the participants' legal guardian/next of kin.

## Author Contributions

MB-P-D, CC, JL, MG-B, and GC-B performed survey and data collection. MG-B, MB-P-D, CC, and GC-B carried out materials and methods and manuscript writing. MB-P-D and VG carried out statistical analysis. MG-B, GC-B, MB-P-D, CC, VG, JL, and BR designed the research and project. All authors contributed to the article and approved the submitted version.

## Conflict of Interest

The authors declare that the research was conducted in the absence of any commercial or financial relationships that could be construed as a potential conflict of interest.

## Publisher's Note

All claims expressed in this article are solely those of the authors and do not necessarily represent those of their affiliated organizations, or those of the publisher, the editors and the reviewers. Any product that may be evaluated in this article, or claim that may be made by its manufacturer, is not guaranteed or endorsed by the publisher.
